# Liver involvement in patients with Gaucher disease types I and III

**DOI:** 10.1016/j.ymgmr.2019.100564

**Published:** 2020-01-07

**Authors:** Rodrigo Tzovenos Starosta, Filippo Pinto e Vairo, Alícia Dorneles Dornelles, Suélen Porto Basgalupp, Marina Siebert, Maria Lúcia Alves Pedroso, Carlos Thadeu Schmidt Cerski, Mário Reis Álvares-da-Silva, Ida Vanessa Doederlein Schwartz

**Affiliations:** a Graduate Programme in Genetics and Molecular Biology, Universidade Federal do Rio Grande do Sul (UFRGS), Porto Alegre, RS, Brazil; b School of Medicine, UFRGS, Porto Alegre, Brazil; c Center for Individualized Medicine, Mayo Clinic, Rochester, MN, USA; d Department of Clinical Genomics, Mayo Clinic, Rochester, MN, USA; e Graduate Programme in Medical Sciences, UFRGS, Porto Alegre, RS, Brazil; f BRAIN Laboratory, Hospital de Clínicas de Porto Alegre (HCPA), Porto Alegre, RS, Brazil; g Unit of Laboratorial Research, Experimental Research Centre, HCPA, Porto Alegre, RS, Brazil; h Graduate Programme in Gastroenterology and Hepatology, UFRGS, Porto Alegre, Brazil; i Graduate Programme in Internal Medicine, Universidade Federal do Paraná (UFPR), Curitiba, Paraná, Brazil; j Gastroenterology Service, Complexo Hospital de Clínicas de Curitiba, UFPR, Curitiba, Paraná, Brazil; k Surgical Pathology Service, HCPA, Porto Alegre, RS, Brazil; l Gastroenterology Service, HCPA, Porto Alegre, RS, Brazil; m Clinical Research Center, HCPA, Porto Alegre, RS, Brazil

**Keywords:** Gaucher disease, Enzyme replacement therapy, Liver steatosis, Hemosiderosis, Biopsy, large-core needle, Cholelithiasis

## Abstract

**Background & aims:**

Gaucher disease (GD) is a multisystemic disease. Liver involvement in GD is not well characterised and ranges from hepatomegaly to cirrhosis and hepatocellular carcinoma. **We aim to** describe, and assess the effect of treatment, on the hepatic phenotype of a cohort of patients with GD types I and II.

**Methods:**

Retrospective study based on the review of the medical files of the Gaucher Reference Centre of the Hospital de Clínicas de Porto Alegre, Brazil. Data from all GD types I and III patients seen at the centre since 2003 were analysed. Variables were compared as pre- (“baseline”) and post-treatment (“follow-up”).

**Results:**

Forty-two patients (types I: 39, III: 3; female: 22; median age: 35 y; enzyme replacement therapy: 37; substrate reduction therapy: 2; non-treated: 3; median time on treatment-MTT: 124 months) were included. Liver enzyme abnormalities, hepatomegaly, and steatosis at baseline were seen in 19/28 (68%), 28/42 (67%), and 3/38 patients (8%), respectively; at follow-up, 21/38 (55%), 15/38 (39%) and 15/38 (39%). MRI iron quantification showed overload in 7/8 patients (treated: 7; MTT: 55 months), being severe in 2/7 (treated: 2/2; MTT: 44.5 months). Eight patients had liver biopsy (treated: 6; MTT: 58 months), with fibrosis in 3 (treated: 1; time on treatment: 108 months) and steatohepatitis in 2 (treated: 2; time on treatment: 69 and 185 months). One patient developed hepatocellular carcinoma.

**Conclusions:**

GD is a heterogeneous disease that causes different patterns of liver damage even during treatment. Although treatment improves the hepatocellular damage, it is associated with an increased rate of steatosis. This study highlights the importance of a follow-up of liver integrity in these patients.

## 1 Introduction

Gaucher disease (GD) (OMIM #230800, #230900 and #231000) is an autosomal recessive disorder most frequently caused by biallelic pathogenic variants in the *GBA1* gene that codes for glucocerebrosidase (GCase). The impaired activity of GCase causes glucosylceramide (GlcCer) to build up into the lysosomes of the reticuloendothelial system cells, mainly macrophages that become engorged and dysfunctional being thus called “Gaucher cells” [1]. The incidence of GD ranges between 1:50,000 and 1:100,000 in the general population, and is about 1:855 in the Ashkenazi Jewish population [2]. GD is broadly categorised in three types, according to neurological manifestations: type I, or “non-neuronopathic”; type II, or “acute neuronopathic”; and type III, or “chronic neuronopathic”.

The manifestations of GD are multisystemic with a complex pathophysiologic process that arises from the infiltration of organs by Gaucher cells, the low-grade inflammation promoted by cells whose intracellular signalling is disrupted by the accumulation of GlcCer [3,4], and other factors such as aberrant complement activity [5,6] and dysfunctional autophagy [7,8]. The main signs and symptoms of GD include hepatosplenomegaly, anaemia, thrombocytopenia, bone deformities and pain, osteonecrosis, restrictive pulmonary disease, and neurological compromise in patients with GD type II and III [1,2] which cause significant impairment in life quality and reduction of life expectancy [9,10]. Treatment of GD is currently available in two modalities: enzyme replacement therapy (ERT) and substrate reduction therapy (SRT). The former is the most established treatment, consisting in the fortnightly infusion of recombinant GCase which is uptaken by the macrophages' lysosomes, decreasing the GlcCer build-up [1,2,11]. Imiglucerase (Sanofi Genzyme Corporation, Cambridge, MA, USA), taliglucerase alfa (Protalix Biotherapeutics, Carmiel, Israel), and velaglucerase alfa (Takeda Pharmaceutical Company, Tokyo, Japan) are the currently available enzymes with no detectable difference in efficacy or safety profile known between them [1,12, [13], [14], [15], [16]]. SRT is administered orally once or twice daily and works decreasing the production of GlcCer which consequently decreases its storage [17]. The currently SRT FDA-approved compounds are miglustat and eliglustat. ERT and/or SRT are not indicated for GD type II patients.

The extent of liver damage in GD is still subject of debate – first reports were limited to hepatomegaly, however it is currently known that patients are at increased risk for focal fibrosis, cholelithiasis, steatosis, haemosiderosis, overt cirrhosis, and hepatocellular carcinoma (HCC) [18,19]. Recent studies [20,21] have shown that liver stiffness is increased in a large proportion of patients with GD, suggesting that fibrosis may be a pervasive process even in patients with apparent controlled disease, and also that it is correlated to disease severity, making it an important cause of morbidity to be addressed in this population.

In this study, we aimed at characterising the liver involvement in a cohort of patients with GD type I and III, and the effect of ERT/SRT on those variables.

## 2 Methods

This is a retrospective study, based on the review of the medical records of the GD types I and III patients followed at the Gaucher Reference Centre of the Hospital de Clínicas de Porto Alegre, Brazil (GRC-HCPA) from 2003 to 2018. HCPA is a public, university hospital located in Southern Brazil. Inclusion criteria were: a) having biochemical or genetic diagnosis of GD; b) not having any other primary liver disease, as determined by clinical and laboratory features and serological screening for hepatitis B and C.

At the GRC-HCPA, patients have regular appointments every 3–4 months and most exams are made in an annual basis unless an acute event prompts a more frequent evaluation. The following exams were performed at baseline for most patients: complete blood count, chitotriosidase activity, aspartate-transaminase (AST), alanine-transaminase (ALT), and abdominal ultrasonography (US). The following exams are performed yearly: AST, ALT, γ-glutamyltransferase (γGT), direct bilirubin (DB), indirect bilirubin (IB), prothrombin time, alkaline phosphatase, total and fractional cholesterol, triglycerides, serum creatinine, blood urea, calcium, phosphorus, US, serum protein electrophoresis, serum immunoglobulins, transferrin saturation/iron-binding capacity, and serum iron. The following exams are performed every three months: complete blood count, serum ferritin, and chitotriosidase activity. All patients are tested for serological markers of viral hepatitis at initiation of treatment and again according to clinical indication. Alpha-foetoprotein (AFP) is not ordered for patients without cirrhosis due to its dubious efficacy as a screening test for hepatocellular carcinoma [22]. The presence of hepatomegaly was ascertained by US or by physical exam (when US was not available). The presence of steatosis was assessed by US. Elastography for fibrosis assessment is not routinely performed. Other exams are performed according to clinical indication [23]. All patients had genotyping of *GBA* and *HFE* by next-generation sequencing.

Immunological and iron metabolism findings of our cohort have already been described by Vairo et al. [24] and Koppe et al. [25], respectively.

Statistical analyses were performed using the SPSS software (IBM Inc., v.18); for comparison of frequencies of categorical variables, the χ^2^ test was used. Patients were compared regarding the findings before the onset of treatment (“baseline” data points) and during treatment until last follow-up (“follow-up” data points). Findings were considered abnormal at baseline or at follow up if altered in at least two measurements for each datapoint, or one measurement when it was the only one available.

## 3 Ethics statement

This study was approved by the Institutional Review Board of HCPA (CEP/HCPA), Porto Alegre, RS, Brazil (projects #13–0537 and #15–0083). All studies were conducted according to the Declaration of Helsinki. Written informed consent was obtained from all subjects or, when <18 years-old, from their parents.

## 4 Results

### 4.1 Subjects

Forty-two patients were included (*n* = 39, type I; n = 3, type III; female = 22; median time on treatment: 124 months). One patient with GD type I (pt 26D) was excluded from the follow-up data analysis due to diagnosis of active chronic hepatitis B. One patient with GD type I (pt 26A) had serological evidence of spontaneously cured hepatitis B. No other patients had signs of other liver diseases, such as drug-related liver injury, autoimmune hepatitis, or viral hepatitis.

No patient had a history of blood transfusions in the past. A total of 36 patients had measurements of serum transferrin saturation after treatment; of these, 6 had decreased values and 5 had increased values (Supplementary Table 1). Four patients had used ferrous sulphate supplements in the past, one of them only during pregnancy (Supplementary Table). No patient was homozygous or compound heterozygous for pathogenic variants in the *HFE* gene, ruling out the concomitant diagnosis of *HFE*-associated haemochromatosis (MIM: #235200).

### 4.2 Laboratory findings

Laboratory findings of all patients are shown in Table 1

**Table 1:** Liver enzymes in patients with Gaucher disease.

Patient	Age	Gender	*GBA* Genotype	Sx	Baseline					Time on treatment	Follow-up				
	(y)				AST	ALT	GGT	DB	IB	(months)	AST	ALT	GGT	DB	IB
1A	7	F	N370S/G202R	No	-	-	-	-	-	None	-	-	-	-	-
1B	22	M	N370S/G202R	No	-	-	-	-	-	I(168)	N	N	N	↑	N
2	20	M	L444P/L444P	No	↑	↑	↑	N	N	I(110) T(1) V(81)	↑	↑	↑	N	N
3	20	F	N370S/L444P	No	-	-	-	-	-	None	-	-	-	-	-
4A	22	F	N370S/L444P	No	N	N	N	N	N	I(68)	N	N	N	N	N
4B	36	F	N370S/L444P	No	N	↑	N	N	N	I(19) T(1) I(90)	N	N	N	N	N
5	23	F	N370S/Rec*Nci*I	No	↑	↑	-	-	-	I(184)	N	N	↑	N	N
6	23	F	N370S/L444P	No	↑	↑	↑	N	N	T(34)	↑	N	N	N	N
7	24	M	N370S/N370S	No	-	-	-	-	-	I(131)	N	N	N	↑	N
8	24	F	N370S/N370S	No	N	↑	N	-	-	I(156)	N	N	N	↑	↑
9	25	F	N370S/L444P	No	N	N	↑	↑	↑	I(113)	N	N	N	↑	↑
10	26	M	N370S/R120W	No	N	N	N	N	↑	I(202)	N	N	N	↑	↑
11A	26	M	L444P/L444P	Yes	-	-	-	-	-	I(176)	N	↑	N	↑	N
11B	29	F	L444P/L444P	Yes	-	-	-	-	-	I(175)	N	↑	N	N	N
12	27	M	N370S/c.1328+1G>A	No	N	N	N	↑	N	I(159)	N	N	N	↑	N
13	27	F	N370S/Rec*Nci*I	No	-	-	-	-	-	I(120) T(1) I(6) E(41) I(8) E(16)	N	N	N	N	N
14	27	M	N370S/L444P; c1483G>C	No	↑	↑	N	-	-	I(237) T(13)	↑	↑	N	↑	↑
15	28	F	N370S/L461P; c.1515+1G>T	No	-	-	-	-	-	I(211)	↑	↑	↑	N	N
16	28	M	N370S/L444R	No	-	-	-	-	-	A(31) I(227)	N	↑	N	N	N
17	31	M	N370S/RecNciI	No	-	-	-	-	-	I(109) T(2) I(9) T(80)	↑	↑	↑	N	N
18	33	F	N370S/L444P	No	N	N	-	-	-	M(7) I(115)	N	N	N	N	N
19A	35	M	N370S/L444P	No	↑	N	↑	N	N	I(75)	N	N	N	↑	N
19B	41	M	N370S/L444P	Yes	N	↑	-	↑	↑	I(74) T(3) I(88)	N	N	N	N	↑
19C	52	M	N370S/L444P	No	N	↑	↑	-	-	I(6) T(1) I(81)	N	N	↑	↑	↑
20	36	F	N370S/R163*	No	-	-	-	-	-	I(210)	↑	N	↑	N	N
21	36	F	N370S/RecNciI	Yes	-	-	-	-	-	A(25) I(28) T(3) I(53)	↑	↑	↑	N	N
22A	37	M	N370S/L444P	No	↑	↑	↑	N	N	I(5) T(81)	↑	N	↑	N	N
22B	39	F	N370S/L444P	No	N	N	N	N	N	I(22) T(2) I(14) E(77)	↑	N	N	N	N
23	38	F	N370S/Rec*Nci*I	No	↑	↑	↑	↑	↑	I(38)	↑	N	↑	N	N
24	45	M	N370S/Rec*Nci*I	No	N	N	-	N	N	I(102) T(3) I(29) T(50)	N	N	N	↑	N
25A	48	F	E349K/S366N	No	N	N	↑	N	N	M(11) T(1) I(62)	N	N	N	N	N
25B	52	F	E349K/S366N	No	N	N	N	N	N	M(30) T(14)	N	N	N	N	N
26A	53	M	N370S/Rec*Nci*I	No	N	↑	↑	↑	N	I(89)	N	N	N	↑	N
26B	57	M	N370S/RecNciI	No	↑	↑	N	N	N	I(79)	↑	↑	↑	↑	↑
26C	63	F	N370S/Rec*Nci*I	No	N	N	↑	↑	↑	I(73)	N	N	N	↑	↑
26D	51	F	N370S/Rec*Nci*I	Yes	-	-	-	N	N	I(96) T(5) I(13) T(79)	-	-	-	-	-
27	62	F	N370S/RecNciI	No	N	N	-	-	-	I(124) T(2) I(8) T(81)	↑	↑	↑	N	N
28	62	M	N370S/Rec*Nci*I	Yes	↑	↑	↑	↑	N	I(34) T(2) I(90)	↑	↑	↑	↑	↑
29A	64	M	N370S/N370S	No	N	N	↑	↑	↑	I(10) T(2) I(9) T(81)	N	N	↑	↑	↑
29B	65†	M	N370S/N370S	No	N	N	↑	N	N	I(19) T(2) I(8) T(14)	N	N	↑	N	↑
30	67	F	N370S/L444R	Yes	N	N	N	N	N	I(12) M(25) I(49)	N	N	N	N	N
31	62†	M	N370S/Rec*Nci*I	No	-	-	-	-	-	None	-	-	-	-	-

Values considered to be elevated were so in at least two measurements while on treatment. Patients sharing the same number are siblings, except for patient 26D who is a cousin of patients 26A, 26B, and 26C. Patients 5, 7, 8 , 14, 19A, 19B, 19C, 21, 22A, and 24 have a low adherence to treatment (less than 75% of programmed yearly infusions performed). Sx = splenectomy; M = male; F = female; N = normal; A = alglucerase; I = imiglucerase; T = taliglucerase alfa; V = velaglucerase alfa; E = eliglustat; - = no value available. †age of death. Reference values: ALT < 34 U/L; AST < 33 U/L; GGT < 40 U/L; DB < 0.4 mg/dL; IB < 0.9 mg/dL.

Out of the 28 patients with liver enzymes (AST, ALT, or γGT) data at baseline, 19/28 (68%) had abnormal liver enzymes in at least two measurements. At follow-up, 21/38 (68%) had abnormalities in at least one liver enzyme in at least two measurements. History was positive for excessive alcohol intake in two patients (19B and 26B).

Serum transferrin saturation, immunoglobulins, and serum protein electrophoresis results during treatment can be found in the supplementary table. Immunoglobulin measurements and serum protein electrophoresis results were available for 36 patients during treatment; of these, 26 had an abnormal serum immunoglobulin measurement at least twice and 20 had increased γ-globulins in serum electrophoresis at least twice.

### 4.3 Liver ultrasound findings

Liver US reports were available from 39 patients (baseline = 39; follow-up = 38) (Table 2, Table 3). Hepatomegaly was present in 28/42 (67%) of patients at baseline and in 15/38 (39%) of patients at follow-up.

**Table 2:** US findings from GD patients at baseline.

Patient	Age (y)	BMI	Steatosis	Hepatomegaly	Cholelithiasis	Ferritin (ng/dL)	MetS^a^	Liver biopsy
1A	7	15.9	No	No	No	378	No	No
1B	8	16.1	No	Yes	No	–	No	No
2	1	16.9	No	Yes	No	–	No	No
3	15	17.3	Yes	No	No	174.6	No	No
4A	16	21.8	No	No	No	284.8	No	No
4B	26	22.7	Yes	Yes	Yes	328.5	No	No
5	8	15.1	No	Yes	No	–	No	No
6	20	22.6	No	Yes	No	219.3	No	Yes
7	6	14.6	No	Yes	No	–	No	No
8	7	12	No	Yes	No	97.3	No	No
9	17	18.3	No	Yes	No	166	No	No
10	9	14.5	No	Yes	No	–	No	No
11A	–	14.1	–	Yes	No	–	No	No
11B	–	–	–	Yes	No	–	No	No
12	12	17.3	No	No	No	–	No	No
13	14	23	No	No	No	–	No	No
14	6	16.2	No	Yes	No	–	No	No
15	11	12.5	No	Yes	No	–	No	No
16	–	15.2	–	Yes	No	–	No	No
17	24	16.8	No	Yes	No	–	No	No
18	17	21.6	No	Yes	No	–	No	No
19A	39	30.2	No	Yes	Yes	758.3	No	No
19B	28	22.2	No	Yes	No	–	No	No
19C	43	26.5	No	No	No	951.8	No	No
20	18	21.9	No	Yes	No	–	No	No
21	13	16.6-	No	Yes	No	–	No	No
22A	30	24	No	Yes	No	469.6	No	No
22B	29	23.2	No	Yes	No	213.3	No	No
23	34	25.3	No	No	No	835	Yes	Yes
24	22	24.3-	No	No	No	–	No	No
25A	42	23.4	No	Yes	Yes	754.2	No	No
25B	43	30.2	Yes	Yes	Yes	860.7	No	No
26A	44	25	No	No	No	811	No	No
26B	50	31.4	No	No	No	1409	Yes	No
26C	57	23.5	No	No	No	1593	No	No
26D	34	19.3	No	Yes	No	–	No	No
27	44	–	No	Yes	No	–	No	No
28	52	24	No	Yes	No	3392	Yes	Yes
29A	55	28.5	No	No	Yes	1698	Yes	No
29B	61	27.2	No	No	Yes	778.2	No	No
30	60	29.8	No	Yes	No	1972	Yes	No
31	62	17.7	No	No	No	1343	No	No

y = years-old; US = ultrasonography; BMI = body-mass index; MetS = metabolic syndrome. Ferritin RV <150 ng/dL for women, <300 ng/dL for men.

a Metabolic syndrome is defined as the presence of at least three of the following: obesity, high triglycerides level, increased blood pressure, and elevated fasting blood glucose (reduced HDL level was not considered as a criterion because it is a feature of GD).

**Table 3:** US from GD patients at follow-up.

Patient	Age (y)	Time on treatment (months)	Steatosis	Hepatomegaly	Cholelithiasis	BMI	Ferritin (ng/dL)	MetS during treatment^a^	Liver biopsy during treatment
1A	–	T(7)	–	No	–	17.4	308	No	No
1B	20	I(168)	No	No	No	28.6	485	No	No
2	20	I(110) T(1) V(81)	Yes^b^	Yes	Yes	21.7	254	No	No
3	–	–	–	–	–	–	–	–	–
4A	–	–	–	No	No	24.4	312.2	No	No
4B	36	I(19) T(1) I(89)	No	Yes	Yes	25.9	57.3	No	No
5	22	I(177)	No	No	No	22.7	84.7	No	No
6	23	T(33)	No	No	No	22.4	172.6	No	No
7	17	I(68)	No	No	No	19.2	885.3	No	No
8	25	I(156)	Yes^b^	Yes	No	20.5	508.3	No	No
9	26	I(112)	No	No	No	22.7	94.6	No	No
10	25	I(200)	No	Yes	No	24.7	547	No	No
11A	26	I(174)	Yes	Yes	Yes	18.8	242.6	No	No
11B	28	I(146)	No	Yes	No	27.8	285.9	No	No
12	25	I(126)	No	Yes	No	23.1	546.3	No	No
13	27	I(120) T(1) I(6) E(41) I(8) E(9)	No^c^	No	Yes	28.8	611.6	No	Yes
14	23	I(189)	No	Yes	No	24.9	619.9	No	No
15	29	I(216)	Yes^b^	Yes	No	26.6	311.1	No	No
16	25	A(31) I(222)	No	No	No	20.6	569.8	No	No
17	29	I(109) T(2) I(9) T(55)	No	No	No	30.6	325.8	No	No
18	33	M(7) I(111)	No	Yes	Yes	25.3	278.8	Yes	No
19A	45	I(67)	Yes^b^	No	Yes	32	66.5	No	No
19B	34	I(71)	No	No	No	27.8	585.3	No	No
19C	51	I(6) T(1) I(80)	Yes^b^	Yes	No	28.3	1084	Yes	No
20	36	I(207)	No	No	Yes	25.6	574.2	No	No
21	32	A(25) I(28) T(3) I(4)	No	No	No	22.7	415.7	No	No
22A	36	I(5) T(65)	No	Yes	No	26.8	463.8	No	No
22B	40	I(22) T(2) I(14) E(70)	Yes	No	No	30	32	Yes	No
23	38	I(35)	Yes^b^	No	Yes	30.7	427.2	No	No
24	44	I(102) T(3) I(29) T(38)	No	Yes	No	28.7	247.9	No	No
25A	48	M(11) T(1) I(55)	Yes	No	Yes	25.8	611.9	No	Yes
25B	51	M(30) T(10)	Yes	No	Yes	31.9	1053	No	Yes
26A	52	I(80)	No	No	No	24.1	480.6	No	No
26B	58	I(76)	Yes	No	No	32.2	1457	Yes	Yes
26C	62	I(66)	No	No	No	21.6	624.8	No	No
27	61	I(124) T(2) I(8) T(71)	Yes	No	No	26.6	1052	Yes	No
28	62	I(34) T(2) I(88)	No	No	No	22.3	543.2	No	Yes
29A	65	I(10) T(2) I(9) T(81)	Yes	No	Yes	34.5	670	Yes	No
29B	65	I(19) T(2) I(8) T(14)	Yes	Yes	Yes	29.7	686	Yes	No
30	67	I(12) M(25) I(47)	No	Yes	No	30.5	1103	Yes	Yes
31	–	–	–	–	–	–	–	–	–

y = years-old; BMI = body mass index; I = imiglucerase; T = taliglucerase alfa; V= = velaglucerase alfa; E = eliglustat; A = alglucerase; M = miglustat.

a Metabolic syndrome is defined as the presence of at least three of the following: obesity, high triglycerides level, increased blood pressure, and elevated fasting blood glucose (reduced HDL level was not considered as a criterium because it is a feature of GD).

b Steatosis regressed within two years of US detection.

c Steatosis at liver biopsy only.

Steatosis was present in 3/39 (8%) of patients at baseline and in 15/38 (39%) at follow-up. In 6 patients, there was regression of steatosis within 2 years of US detection. Of these, none had any significant change in body-mass index (BMI) but two had changes in the ERT regimen (for patient 15, there was an increase in the imiglucerase dosage from 45 IU/Kg to 60 IU/Kg; for patient 19C, there was a switch from taliglucerase alfa to imiglucerase). Twelve out of the 16 patients (75%) with steatosis were overweight or obese, with 4 patients (two whose steatosis regressed, one that maintains the finding, and one that denied treatment and further follow-up) having a normal BMI. A significant difference was found between the frequency of overweight/obesity in patients with and without persistent steatosis (77.8% *vs* 40%, *p* = .047, Pearson's χ^2^). Blood lipid levels were available for 7 of the 9 patients (78%) with non-regressing steatosis during treatment. All 7 patients had dyslipidaemia (four with high triglycerides, three with high total cholesterol and LDL, and five with low HDL). Levels were available for 31 patients without non-regressing steatosis – of these, 28 (90.3%) had dyslipidaemia (10 with high triglycerides, 5 with high total cholesterol and LDL, and 25 with low HDL). No significant difference was found between patients with and without non-regressing steatosis and the presence of dyslipidaemia (*p* = .814, Pearson's χ^2^).

Twelve patients in the cohort had cholelithiasis, and 7 of them underwent cholecystectomy (pts. 13, 18, 19A, 20, 23, 25A, and 25B). Patient 23 had cholecystectomy before initiation of treatment for GD. Eight out of the patients with cholelithiasis were overweight or obese, but no significant difference in the prevalence of overweight/obesity was found between the patients with and without cholelithiasis (66.7% *vs* 40.7%, *p* = .135, Pearson's χ^2^).

Other US findings observed in the cohort were: cysts, haemangioma, solid nodule compatible with an adenoma or a haemangioma, portal hypertension that resolved with initiation of ERT, and cirrhosis with HCC. The two cysts of unknown diagnosis were present in a pair of brothers with GD type I who also had steatosis (pts 29A and 29B). The older brother passed away at the age of 65 due to multiple myeloma. The cyst in the younger brother, now aged 65, is 5 mm in diameter and is stable since it was diagnosed 2 years ago. The patient with cirrhosis (pt 28) is a 62-year-old male splenectomised GD type I patient described elsewhere ^26^.

### 4.4 Magnetic resonance iron quantification

Liver iron quantification by magnetic resonance had been performed in 7 patients with GD type I on treatment with ERT (Table 4). Iron overload was observed in 6/7 (85%) patients, ranging from 50 to 280 μmol/g (reference value (RV): <36). All the patients with iron overload had high ferritin values, ranging from 244 to 3011 ng/mL. Two patients had a high level of iron overload (>79 μmol/g [27]) – one was a 55-year-old male patient whose MRI showed a concentration of 280 μmol/g of iron in the liver, with ferritin at 1813 ng/mL and transferrin saturation at 47.3% (RV 20–45%), steatosis, and a heterozygous c.845G>A, p.Cys282Tyr variant in *HFE* gene. The other patient with high iron levels (210 μmol/g) is a 64-year-old female who had ferritin at 3011 ng/dL and low transferrin saturation at 17.4%. There were no signs of steatosis and she doesn't harbour any pathogenic variant in *HFE* gene.

**Table 4:** Patients screened for hepatic iron overload with magnetic resonance.

Patient	Age (y)	Gender	*HFE* genotype	Ferritin (ng/dL)	Transferrin saturation	Treatment at exam	Time on treatment (months)	Iron concentration (μmol/g)
10	26	M	p.Cys282Tyr/wt	525	–	I	I(196)	70
15	24	F	wt/wt	298.4	30.7%	I	I(160)	50
23	34	F	p.His63Asp/wt	835	25%	None	–	5
25A	44	F	p.His63Asp/wt	937.1	50.8%	I	M(11) T(1) I(8)	55
26B	55	M	p.Cys282Tyr/wt	1813	47.2%	I	I(55)	280
27	59	F	wt/wt	347	44%	T	I(124) T(2) I(8) T(41)	65
30	63	F	wt/wt	3011	17.4%	M	I(12) M(22)	210

All patients are type I. Ferritin and transferrin saturation values given are approximately from the time of the MR iron quantification. Reference values: ferritin (males) <300 ng/mL; ferritin (females) < 150 ng/mL; transferrin saturation 25–45%; iron concentration < 6 μmol/g. y = years-old; *HFE* = homeostatic iron regulator gene; F = female; M = male; wt = wild-type; I = imiglucerase; T = taliglucerase; M = miglustat.

### 4.5 Liver biopsy

**Fig. 1:**
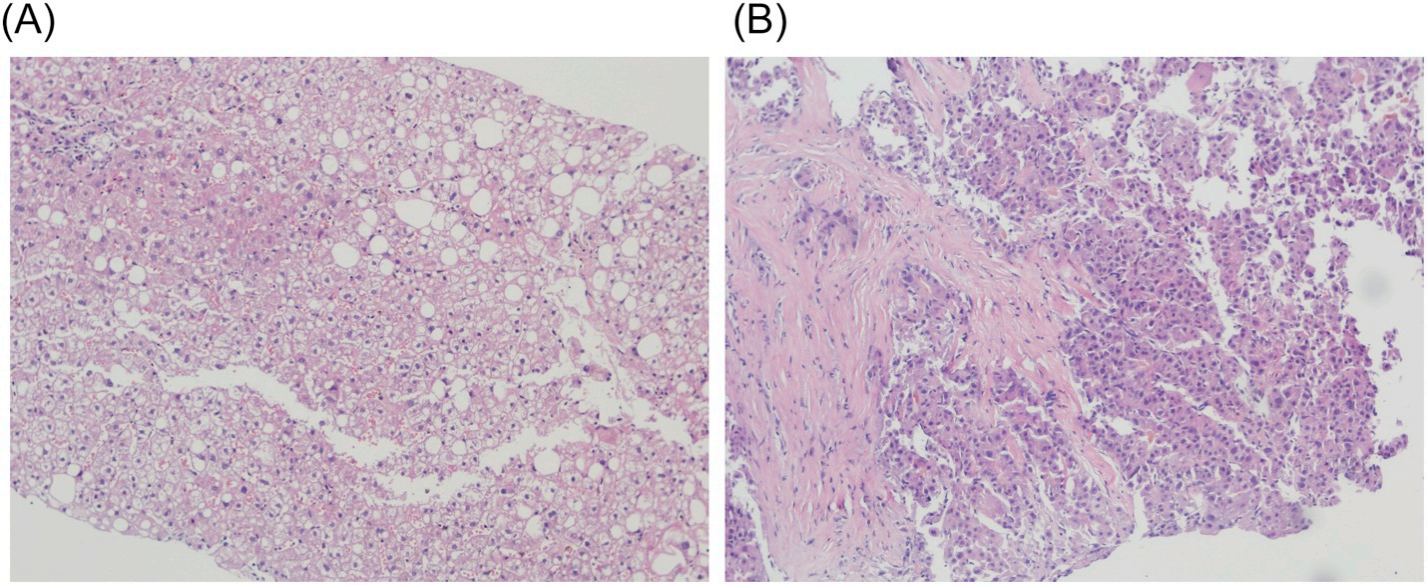
A: Haematoxylin and eosin, 200× magnification. Liver biopsy of patient 26B showing macrovesicular steatosis in approximately 5% of hepatocytes, as well as a small focus of mixed inflammation (upper left corner). Fig. 1B: Haematoxylin and eosin, 400× magnification. Liver biopsy of patient 28 showing thick bridging fibrosis characteristic of cirrhosis, as well as substitution of the local hepatic parenchyma by moderately differentiated hepatocellular carcinoma cells.

Six patients with GD type I had a liver biopsy done (Fig. 1; Table 5) when on-treatment. One patient was found to have Gaucher cells in the liver parenchyma; One patient had atypical Gaucher cells in a cirrhotic parenchyma with severe iron overload in hepatocytes and Kupffer cells, and, in a subsequent biopsy, a moderately differentiated HCC [26]. Two patients who have had mild to moderate steatosis on ultrasound had a biopsy confirming macrovesicular steatosis – one also with evidence of cholestasis and a few foci of inflammation, and the other with mild haemosiderosis. Two patients had steatohepatitis with mild activity: a 27-year-old female with a BMI of 28.8 Kg/m^2^ who did not show any sign of steatosis in the ultrasound, had normal serum blood glucose and lipid profile except for a low HDL (which is expected in GD) and that was on SRT with eliglustat at the time of the biopsy; and a 58 year-old man had moderate-to-severe haemosiderosis of hepatocytes and Kupffer cells, elevated triglycerides and total and LDL cholesterol, and low HDL, albeit a normal blood glucose, and signs of steatosis in the liver ultrasound, and that was on ERT at the time of biopsy.

**Table 5:** Findings in the liver biopsy.

Patient	Age (y) at biopsy	Treatment at biopsy (IU/Kg)	Time on treatment (months)	Inflammation	Steatosis	Siderosis	Gaucher cells	Fibrosis	Other findings
6	20	None	–	No	No	No	Yes	Perisinusoidal	No
13*	27	E	I(120) T(1) I(6) E(41) I(8) E(9)	Steatohepatitis		No	No	No	No
23*	34	None	–	No	No	No	Yes	Bridging	No
25A*	44	I [30]	M(1) T(1) I(14)	Mild	Mild	Mild	Yes	No	Cholestasis
25B*	49	M	M(11)	No	Mild	Mild	No	No	No
26B	56	I [30]	I(69)	Steatohepatitis		Severe	No	No	No
28	60	I [15]	I(34) T(2) I(72)	No	No	Severe	Yes	Cirrhosis	HCC
30*	63	M	I(12) M(22)	Mild	No	Moderate	Yes	No	Nuclear glycogenosis

y = years-old; Sx = splenectomy; E = eliglustat; I = imiglucerase; M = miglustat; HCC = hepatocellular carcinoma. In patient 28, HCC was noted only in a second biopsy, performed 9 months after the first one. *patients with normal liver enzymes; see Table 1.

Two patients with GD type I underwent liver biopsy before treatment initiation. A 34-year-old female's biopsy showed bridging (stage 3) fibrosis and scattered Gaucher cells; in the other, a 20-year-old woman, peri-sinusoidal fibrosis was noted together with high serum AST, ALT, and γGT, and a normal liver ultrasound.

## 5 Discussion

For the past few decades, the liver involvement in GD has become subject of great importance in the patients' management. It is now recognised that hepatomegaly is only one of the manifestations of hepatic compromise in GD, and more attention is needed to all the possible comorbidities that may arise from it. In our cohort, we observed that a significant number of patients have mildly increased markers of hepatic and biliary damage before the treatment initiation and throughout the clinical follow-up, indicating that a low-grade process of liver damage is not fully corrected by the treatment. This finding resembles the study by James et al. from when effective treatment for GD was not available [28], in which most patients with GD had mild-to-moderate transaminase elevations. In more recent cohorts, these alterations have also been found in a lesser proportion of patients [20,21]. However, the impact of these alterations is still unclear. Nascimbeni et al. have shown that levels of liver enzymes are not correlated with liver fibrosis [20]. The contribution of chronic liver damage to the development of other complications such as iron deposition, since chronic hepatitis and liver disease are strongly associated with hemosiderosis [29], has not been fully explored to date. The high frequency of patients with elevations in γGT may also be related to the known biliary alterations caused by GD [30] such as changes in bile composition, increased incidence of cholelithiasis, or with the chronic inflammatory process that happens in the disease [3,4,24] causing biliary damage.

A significant proportion of patients had bilirubin elevations, both before and during treatment. Most elevated bilirubin values corresponded to direct bilirubin, which points toward a biliary cause rather than overproduction (*e.g.*, haemolysis). It is difficult to establish a clinical significance of this finding, It is known that GlcCer and glucosylsphingosine (GlcSph) [31,32] interact with a series of transporters of the ABC (ATP-binding cassette) family, including ABCB1 [33]; It is also known that the bile of patients with GD is different than in the general population, being composed of lower total lipid concentration and, in some patients, high relative concentration of sphingolipids [30]; and finally, that ABC transporters such as ABCB1 are capable of transporting GlcCer and GlcSph [34] across cell membranes, and are modulated by these complex lipids [33]. ABCB1 is localized at the canalicular membrane contributing to the bile formation and xenobiotic excretion [35] – it is possible that, due to ABC-mediated efflux, the higher levels of GlcCer present in bile [36] lead to canalicular disturbances that may cause an impaired flow of bilirubin, leading to the slightly high levels of DB observed.

Iron homeostasis is being increasingly recognised as a key factor of GD's pathogenesis [25]. In a recent article by Lefèbvre et al. [37], it was reported that a local overstimulation of hepcidin related to the lower enzymatic activity of GCase causes iron to be sequestered within macrophages and other cell types, leading to a lower level of free iron, transferrin-bound iron and a higher production of ferritin by the liver. In our study, we observed that several patients with GD have high hepatic iron levels as measured by magnetic resonance, two of the tested patients with levels consistent with severe iron overload – whilst in one patient it may be caused by other risk factors such as alcoholism, steatohepatitis, and a pathogenic *HFE* variant, in the other patient the only obvious risk factor is obesity, and the low transferrin value with exceedingly high ferritin confirm the predictions by Lefèbvre et al. Other studies have observed increase liver iron concentration in GD patients [38], with a positive correlation with serum ferritin concentration. On liver biopsy, positive iron staining has been described extensively [28,39] both in Kupffer cells and in hepatocytes, similar to what was observed in our cohort. Data on pre- (median = 19%, *n* = 8 patients) and post-treatment (median = 28%, *n* = 13 patients) values for serum transferrin saturation in this cohort have been described by Koppe *et al* [25], with no significant difference (*p* = .138).

The main ultrasound finding in our cohort was steatosis, with predominance in overweight/obese patients. Our findings differ from the Israeli cohort, which has a much lower prevalence of fatty liver and a higher prevalence of focal lesions [40]. In the Israeli study, 500 patients were evaluated by US, of which 39 had ultrasonographic evidence of hepatic disease – of these, two-thirds were on ERT and one-fourth was splenectomised. ERT is a potent inducer of weight gain due to slowing the increased basal metabolic rate of patients before treatment [19,41]; thus, it may be difficult to establish whether the high prevalence of steatosis is a manifestation of GD itself, a complication of its treatment, or a comorbidity. A significant proportion of our patients had dyslipidaemia, which indicates that metabolic syndrome may play a role as a confounder in the development of steatosis in these patients [42]. Remarkably, a young patient being treated with eliglustat that had a hepatic biopsy done during cholecystectomy was diagnosed with steatohepatitis, regardless of having no signs of steatosis. This case raises two questions: whether ultrasound can be relied upon as a mean of screening for liver disease in GD patients; and whether steatohepatitis may be a manifestation of GD, since the only known risk factor that the patient had for steatohepatitis (a BMI of 28.8 Kg/m^2^) is hardly considered enough for a sole causal factor; and, as the blood glucose and lipid levels of this patient were normal except for a low HDL, which is a marker of GD, dyslipidaemia and metabolic syndrome are not strongly suspected. Another possible cause for the steatohepatitis in this patient could be what is becoming known as “lean fatty liver disease” – that is, non-alcoholic steatosis (NAFLD) or steatohepatitis (NASH) in patients with few or no risk factors for such [43]. Although in the classical definition of “lean NASH” the patient's BMI is normally <25 Kg/m^2^ [43], despite some authors advocating for the use of a BMI of <30 Kg/m^2^ in Western populations [44], it is expected that patients with “non-lean NASH” are male, of older age, and have hypertension, insulin resistance, or hypercholesterolaemia - none of which is present in this patient [43]. It is speculated that lean NASH arises from “metabolic obesity” in non-obese people, which is reflected by the higher distribution of fat to the visceral intraabdominal organs [44,45], along with classical risk factors such as insulin resistance and hypercholesterolaemia [44] – none of which were present in this patient – and genetic predisposition due to polymorphisms in genes associated with lipid metabolism [44,46].

Liver fibrosis is shown to be increased in a significant proportion of patients [20], especially in those who were splenectomised [38], and it is a major risk factor for HCC [39]. Liver fibrosis is correlated with increased severity of GD [20], although its correlation with biomarkers of disease activity is still controversial [20,38]. In the pre-ERT era, when no specific treatment for GD was available, liver fibrosis was a common finding [28], and often culminated in a massive central area of hypocellular fibrotic tissue [47,48] that led to portal hypertension and other clinical manifestations of cirrhosis [28].

Cholelithiasis is a frequent comorbid process of GD with about 30–45% [49,50] lifetime incidence in these patients. Although the causes for this increased incidence are not completely elucidated, some authors speculate that the excretion of GlcCer in the bile may increase its lithogenicity, predisposing to the formation of gallstones [30,36,49]. In our cohort, we have observed a similarly increased prevalence of cholelithiasis in GD patients compared to the general population, with 12 patients affected in a total of 41.

## 6 Conclusion

In this study, we presented a comprehensive summary of the hepatic manifestations in a well-characterised cohort of patients with GD, showing that several patients have lingering alterations that may indicate a smouldering process of liver damage which is not completely avoided by standard therapy. It is also noticeable that many patients have liver steatosis or steatohepatitis, with a noticeable increase in prevalence during treatment with ERT, but it is still unclear whether it reflects a consequence from the treatment, a feature of the disease, or a coincidental finding.

## Appendix A Supplementary data

Supplementary material Image 1
