# Supplementary material for: Liver involvement in patients with Gaucher disease types I and III
Source: Mol Genet Metab Rep. 2020 Jan 7;22:100564. doi: 10.1016/j.ymgmr.2019.100564 (PMC7026612; doi:10.1016/j.ymgmr.2019.100564)
Supplement: Supplementary file 1 — Supplementary material [file mmc1.docx]

| Patient | Steatosis | Serum transferrin saturation | Iron supplementation | Immunoglobulins | Serum protein electrophoresis |
| --- | --- | --- | --- | --- | --- |
| 1B | No | Normal | No | Normal | Normal |
| 2 | Yes | Low | No | ↑IgE, ↑IgG | ↑γ-globulin |
| 4A | - | - | No | Normal | Normal |
| 4B | No | Low | No | ↑IgA, ↑IgM | Normal |
| 5 | No | Low | No | ↑IgE | ↑γ-globulin |
| 6 | No | Normal | No | ↑IgE | ↑γ-globulin |
| 7 | No | - | No | ↑IgA, ↑IgE, ↑IgM | ↑γ-globulin |
| 8 | Yes | Low | No | ↑IgG | ↑γ-globulin |
| 9 | No | Normal | Yes | Normal | ↑γ-globulin |
| 10 | No | High | No | ↑IgE | Normal |
| 11A | Yes | Normal | No | ↑IgA, ↑IgE, ↑IgG | ↑γ-globulin |
| 11B | No | Normal | Yes | ↑IgE, ↑IgG | ↑γ-globulin |
| 12 | No | High | No | Normal | Normal |
| 13 | Yes | Normal | No | ↑IgG | ↑γ-globulin |
| 14 | No | Normal | No | Normal | Normal |
| 15 | Yes | Normal | No | ↑IgG, ↑IgM | ↑γ-globulin |
| 16 | No | Normal | No | ↑IgE | ↑γ-globulin |
| 17 | No | Normal | No | ↑IgE | Normal |
| 18 | No | Low | No | ↑IgA, ↑IgG | ↑γ-globulin |
| 19A | Yes | Normal | No | Normal | Normal |
| 19B | No | Low | No | ↑IgG, ↑IgM | ↑γ-globulin |
| 19C | Yes | Normal | No | Normal | Normal |
| 20 | No | Normal | Yes* | ↑IgA, ↑IgE, ↑IgG, ↑IgM | ↑γ-globulin |
| 21 | No | Normal | Yes | ↑IgG | ↑γ-globulin |
| 22A | No | Low | No | ↑IgE | Normal |
| 22B | Yes | Low | No | ↑IgE, ↑IgG | Normal |
| 23 | Yes | Normal | No | ↑IgE, ↑IgG, ↑IgM | ↑γ-globulin |
| 24 | No | Normal | No | ↑IgM | ↑γ-globulin |
| 25A | Yes | High | No | ↑IgE, ↑IgM | Normal |
| 25B | Yes | Normal | No | ↑IgA, ↑IgE | ↑γ-globulin |
| 26A | No | Normal | No | ↑IgE | ↑γ-globulin |
| 26B | Yes | High | No | ↑IgE | Normal |
| 26C | No | Normal | No | ↑IgM | Normal |
| 27 | Yes | Normal | No | Normal | Normal |
| 28 | No | High | No | ↑IgA, ↑IgE, ↑IgG | ↑γ-globulin |
| 29A | Yes | Normal | No | Normal | ↑γ-globulin |
| 29B | Yes | Normal | No | ↑IgG^b^ | ↑γ-globulin^b^ |
| 30 | No | Normal | No | Normal | Normal |

Supplementary table 1: Data regarding transferrin saturation, iron supplementation, and markers of inflammation patients during treatment (at follow-up)

^a^Iron supplementation received during pregnancy only. ^b^Patient with multiple myeloma. Reference values: serum transferrin saturation 20-45%; IgA 61-348 mg/dL; IgE <130 mg/dL; IgG 550-1550 mg/dL; IgM 23-259 mg/dL. Values were considered altered when abnormal in at least two measurements during treatment for GD. Paediatric hyperimmunoglobulinaemia in this cohort has been previously described by Vairo *et al*^30^.
